# The High Energy diffraction beamline at the Canadian Light Source

**DOI:** 10.1107/S1600577525001262

**Published:** 2025-03-26

**Authors:** Aly Rahemtulla, Graham King, Ariel Gomez, Narayan Appathurai, Adam F. G. Leontowich, Rielly Castle, Nicholas Burns, Chang-Yong Kim, Beatriz Moreno, Stefan Kycia

**Affiliations:** a Canadian Light Source 44 Innovation Blvd Saskatoon SK S7N 2V3 Canada; b University of Guelph 50 Stone Road E Guelph Ontario Canada; University of Essex, United Kingdom

**Keywords:** pair distribution function, high energy diffraction, high pressure diffraction, Laue monochromator, hard X-ray beamline

## Abstract

The design, performance, and capabilities of the High Energy beamline at the Brockhouse Sector of the Canadian Light Source are described. The beamline uses a single bent silicon wafer as a side-bounce Laue monochromator, using the (111), (422), or (533) *hkl* reflections to access energies ranging from 25 to 90 keV. The cryogenically cooled crystal serves as the only optical element in the beamline providing a simple, convenient, and reliable configuration. The bending provides a vertical focus as small as 20 µm. The flux ranges from 1 × 10^10^ to 1 × 10^13^ photons s^−1^, depending on the energy, with typical pre-monochromator slit settings. A large translation table in the hutch moves to follow the beam as the energy is changed. Data are collected using large area detectors. Common uses include rapid collection of powder diffraction data, penetration of thick samples and devices, high pressure diffraction, and pair distribution function measurements.

## Abstract 2

The performance of the High Energy beamline of the Brockhouse Sector of the Canadian Light Source is described in terms of flux, bandwidth, divergence, and focus of the beam. Its uses include high energy penetrating diffraction, high pressure diffraction, and pair distribution function studies.

## 1. Introduction

High energy X-ray beams produced by synchrotrons enable many types of experiments that are not possible using a laboratory source. The penetration power of these beams coupled with their high flux permits rapid collection of powder diffraction data through thick solid samples or *operando* devices. Access to high *Q* data also allows the short and medium range structure of materials to be probed by means of the pair distribution function. Several synchrotron facilities currently operate successful user programs on high energy diffraction beamlines (Bernasconi *et al.*, 2015 ▸; Chupas *et al.*, 2007 ▸; Connolley *et al.*, 2020 ▸; Dippel *et al.*, 2015 ▸; Drakopoulos *et al.*, 2015 ▸; Isshiki *et al.*, 2001 ▸; Kohara *et al.*, 2007 ▸; Martínez-Criado *et al.*, 2012 ▸, Martínez-Criado *et al.*, 2016 ▸; Mezouar *et al.*, 2005 ▸; Ohara *et al.*, 2018 ▸; Vaughan *et al.*, 2020 ▸; Wright *et al.*, 2020 ▸). This paper describes a new high energy diffraction and scattering beamline located at the Canadian Light Source (CLS).

The Brockhouse X-ray Diffraction and Scattering (BXDS) sector at the CLS is a set of three beamlines powered by two insertion devices that enable a variety of X-ray scattering techniques over a broad range of energies (5–90 keV) to service the scientific communities in Canada and abroad. The High Energy (HE) wiggler beamline is one of the beamlines at BXDS and is the focus of this publication. The HE beamline produces monochromatic X-rays between 25 and 90 keV.

## 2. Beamline design

**Figure 1:**
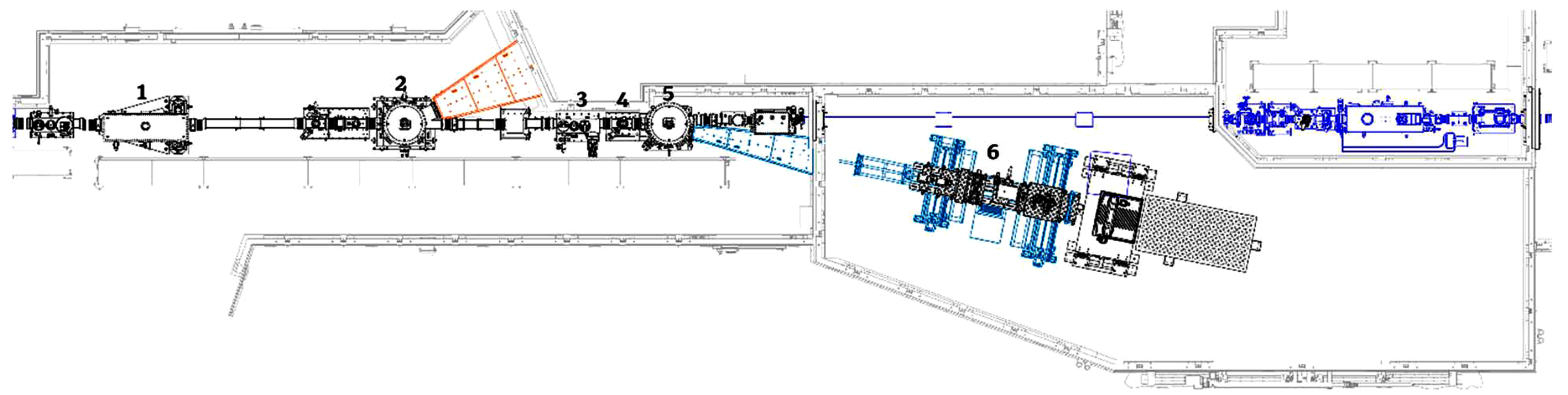
Bird’s eye layout of the BXDS-HE beamline. Optic 1 is a white beam mirror that splits a side portion of the wiggler beam to Optic 2, which is the BXDS-LE monochromator. The central portion of the wiggler beam passes through to Component 3, white beam slits, followed by a removable diamond screen for white beam viewing (Component 4). Optic 5 is the double bent Laue monochromator, which can switch between two crystals. The monochromatic beam is diffracted to the translation table (Component 6), which holds additional slits, sample stage, detector, *etc*.

The CLS is a 2.9 GeV storage ring consisting of 12 straight sections and typically operates in top up mode with a beam current of 220 mA. BXDS occupies Section 4 of the CLS storage ring. An in-vacuum undulator and in-vacuum wiggler share the straight section and are chicaned by 4 mrad. The wiggler beam first passes through a series of masks and filters. The wiggler beam is then split using a long toroidal mirror which reflects the inboard off-axis section of the beam (Fig. 1 ▸). The deflected beam then encounters a monochromator which further deflects the beam outboard to be used at the neighboring Low Energy (LE) wiggler beamline. The selection and design of the wiggler, optics common to both wiggler beamlines, the mirror, as well as the design and performance of the LE beamline have been described previously (Leontowich *et al.*, 2021 ▸).

The central portion of the wiggler radiation fan, which passes by the mirror, then encounters a second filter assembly. This assembly has three filters (from upstream to downstream): 1.8 mm of annealed pyrolytic graphite, 0.41 mm of Al, and 1.6 mm of Al. The filters are clamped in water-cooled copper frames. The first two filters are permanent while the third 1.6 mm Al filter is optional and pneumatically actuated. The graphite strongly attenuates X-rays with energies below ∼5 keV, while Al attenuates most X-rays with energies below ∼12 keV. Together these filters remove X-rays with energies below the operational range of the beamline, which reduces the heat load on the subsequent components. Next, the beam passes through a set of tungsten slits which define the lateral and vertical size of the beam that reaches the monochromator. These can be set as wide as 10 mm × 10 mm but during actual data collection the slits are always set much narrower to reduce the energy bandwidth and beam spread. The horizontal gap is usually narrower than the vertical as it has a larger impact on beam bandwidth. The undulator beam passes by all the wiggler optics unaltered until it reaches its own set of primary optics (Diaz *et al.*, 2014 ▸). This design allows three independently operable beamlines from two insertion devices in a single straight section.

**Figure 2:**
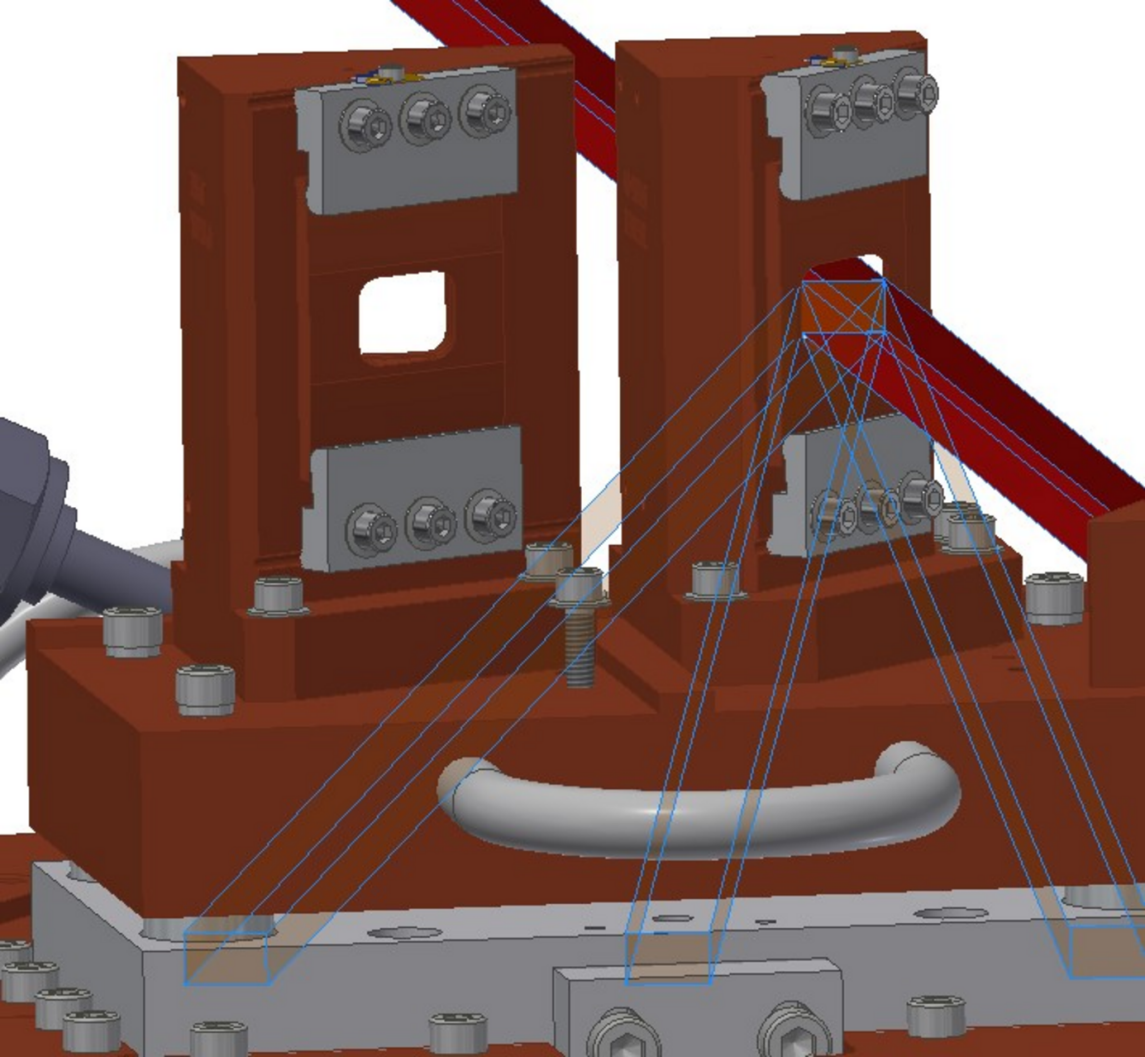
CAD model of the liquid N_2_ cooled crystal carriage of the HE monochromator. There are two carriages that house the Si(111) and the Si(422)/(533) crystals. The carriage is shown without the crystals so that the beam openings can be seen. The red line shows the white beam passing through the (111) opening and hitting the beam stop. The light blue lines show the diffracted monochromatic beam over the angular range it is possible to access by rotation of the stage. The stage can be translated perpendicular to the white beam to switch between crystals.

The monochromator has a bent Laue design which serves the triple purposes of deflecting the beam inboard away from the undulator beam, selecting a particular wavelength, and focusing the beam (Gomez *et al.*, 2018 ▸). Each crystal is precisely bent against a machined block to achieve a specific radius of curvature, optimizing the desired focus. The monochromator carriage is cooled with liquid nitro­gen and contains two rotatable Si single crystal wafers oriented for different Bragg reflections to be accessed on the same rotation plane (Fig. 2 ▸). One crystal is oriented to make use of the (111) reflection while the other is oriented so that the (422) or (533) reflections can be accessed. In general, the (111) crystal is intended to be used for relatively lower energy X-rays (25–37 keV) while the (422)/(533) crystal is meant for higher energy X-rays (39–90 keV). A beryllium window on the downstream end of the monochromator chamber is where the ultra-high vacuum environment of the beamline ends. The distance between the center of the wiggler and the monochromator is 24.54 m.

This monochromator was built in-house, using Huber motorized stages for the crystal alignment on the beam. The silicon wafers were cut to a rectangular shape, etched to relieve surface stress, and clamped from the top and bottom edges to a Ni-plated copper holder with a vertical curvature. This defined the crystal vertical curvature responsible for the beam vertical focusing. The anticlastic bending occurs naturally and is responsible for the beam horizontal focusing. We measured the curvature of the crystals using a Zygo 3D optical profilometer. The measured vertical curvatures of the crystals were very close to the holder’s curvature: 0.37 m and 0.72 m for the lower energy and higher energy crystals, respectively. The large horizontal curvature (over 20 m) was more difficult to control and we experimented with different crystal widths, until obtaining values close to the requirement for horizontal focusing (28–37 m) (Gomez *et al.*, 2018 ▸). We also found that the horizontal curvature was skewed and uneven at different sections of the crystals, which could be the reason for the larger experimental horizontal focus when compared with the simulations.

The single crystal monochromator means the path of the beam is now energy dependent. To enable energy selection, all components downstream of the monochromator, including the experimental hutch components, endstations and detectors must follow the 2θ angle of the monochromator. The HE hutch has a large translation table with two translation rails enabling its motion to match the horizontal 2θ radial direction centered on the monochromator. The translation table can access an effective 2θ range of 6–14°, which defines the accessible energies of each monochromator, leaving only a narrow energy blind spot between 37 and 39 keV. The fixed curvature on the Laue crystals causes the optimum focal position to shift upstream as photon energy increases. The HE beamline table and sample motors are controlled through *EPICS* (EPICS, 2022 ▸) with direct user operation through *SPEC* (Swislow, 1998 ▸) and *Bluesky* (Allan *et al.*, 2019 ▸).

## 3. Beamline performance

The flux was measured using a First Sensor 8 mm thick CsI:Tl diode designed to have 100% absorption within the photon energy range of this beamline. The diode was calibrated at the Biomedical Imaging and Therapy Insertion Device (BMIT-ID) beamline (Gasilov *et al.*, 2024 ▸) at the CLS using a radiometer. The diode readings were characterized using the absolute beam power readings of a radiometer over several energies and filters to accurately characterize the performance at each energy. The diode was then used at the HE beamline to measure the photon flux over the accessible energy range. The tungsten slits were set at 2 mm × 4 mm (horizontal × vertical) as this is the most commonly used setting for actual experiments. If the slits are fully open, the beamline flux will be roughly double the values described below.

**Figure 3:**
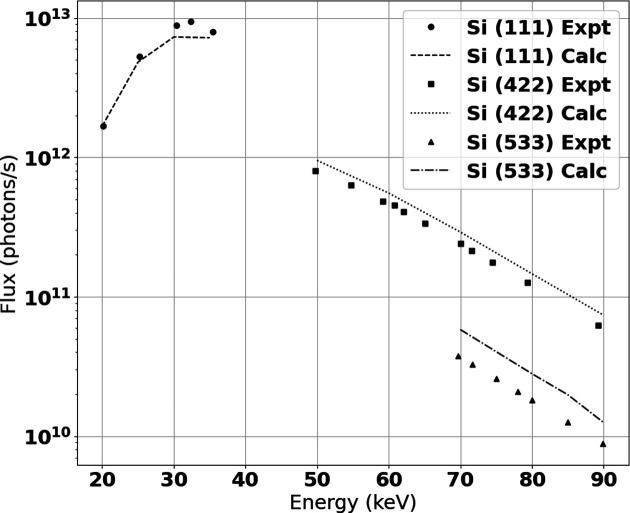
Measured and simulated flux at maximum beam acceptance and 220 mA ring current across all three monochromator *hkl* options.

The measured flux values are plotted in Fig. 3 ▸ as a function of energy and monochromator reflection. A high flux of around 1 × 10^13^ photons s^−1^ is achieved off the (111) reflection for the lower energy range of this beamline. This compares very closely with simulated values calculated through ray tracing, and slightly higher flux is observed in the lower energy range using the (111) *hkl* reflection. It is possible there is variance in the thickness of the Al filters, which would dampen lower energy photons significantly more than higher energies. The Al filters cause the decrease in flux at energies below 30 keV.

The (422) and (533) reflections have a large overlap in accessible energies. The (422) reflection provides a higher flux with a larger bandwidth while, due to the higher Bragg angle, the (533) has a smaller energy bandwidth and a lower flux. The fixed curvature of the (533) crystal will have a vertical focal position further upstream than the (422) (Gomez *et al.*, 2018 ▸). In practice, the (422) is generally used for energies between 55 and 70 keV and the (533) for energies between 70 and 90 keV, since for these energies the focal positions occur at convenient positions on the translation table.

**Figure 4:**
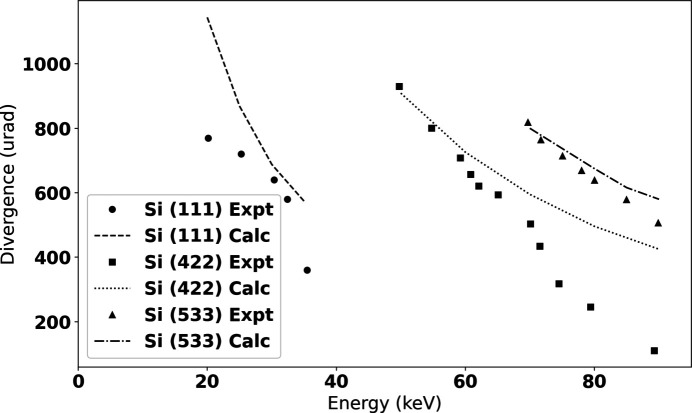
Experimentally determined vertical beam divergence measured using the rocking curves of Si (333) and (777) reflections, over-plotted with the simulated divergences for all three crystals *hkl* reflections.

Energies, bandwidth, and vertical divergence were determined by analyzing rocking curves of the (333) and (777) reflections of a Si wafer, measured using a two-circle Huber 414 stage stack as described by Leontowich *et al.* (2021 ▸). The results are shown in Figs. 4 ▸ and 5 ▸ and agree reasonably well with theoretical values.

**Figure 6:**
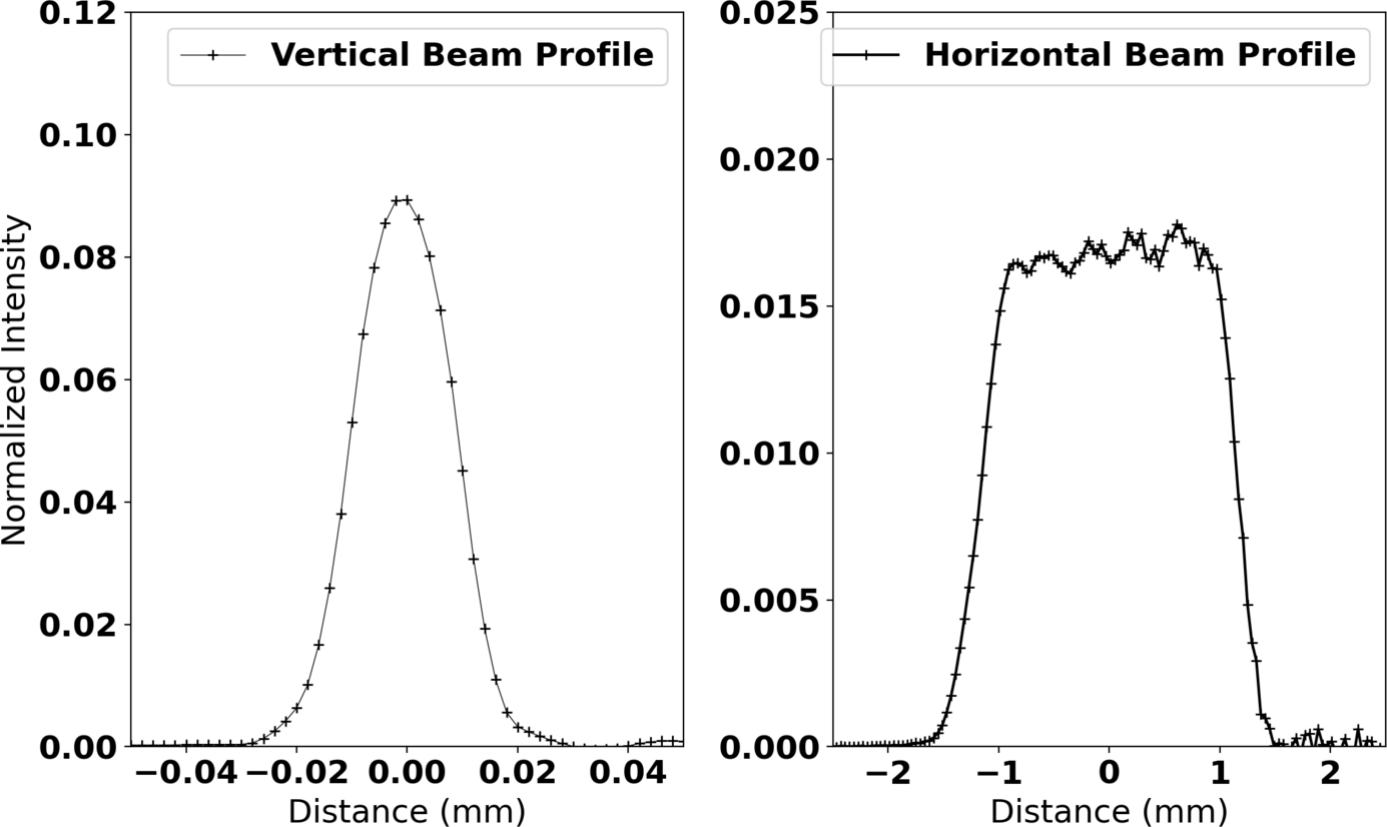
Experimentally determined focused beam profile at the vertical focus position for 60 keV X-rays using the Si (422) monochromator.

Fig. 6 ▸ shows the horizontal and vertical beam profile at the smallest vertically focused position at 59.19 keV using the (422) reflection. The profile is created through the first derivative of a knife edge scan. The beam focuses with a full width at half-maximum (FWHM) of 20 µm vertically, but has a large horizontal FWHM of 2.23 mm. Ray tracing simulations predict a similar minimal vertical focus of 15 µm and a horizontal beam size of 1.6 mm. Similar measurements were taken at 30 keV using the (111) reflection and 80 keV using the (533) reflection. The results are shown in Table 1 ▸. Both vertical and horizontal experimental beam are about 25% larger than calculated. Discrepancies in the actual bending radii in comparison with the designed dimensions could account for these differences.

Simulated values of beam flux, energy bandpass, beam divergence, and beam size at focus have been determined via the X-ray ray tracing software *xrt* (Klementiev & Chernokov, 2014 ▸). The ray tracing simulations were initiated by first reconstructing the beamline layout and defining the wiggler source using the targeted storage ring and wiggler magnet parameters including the electron beam energy and current, the wiggler deflection parameter, and an appropriate energy range to simulate (Cutler *et al.*, 2007 ▸; Gomez *et al.*, 2018 ▸). A minimum of a million rays are generated from the wiggler source to ensure a realistic representation of the beamline. Great care was taken when modeling the monochromator crystals as it is often very difficult to accurately model anti­clastic crystals. The *xrt* software implements the Takagi–Taupin model to include effects of crystal strain and volumetric diffraction (Klementiev & Chernokov, 2023 ▸). The monochromator crystals were built within the simulations according to the targeted radii of curvature and asymmetry angle. No crystal mosaicity is considered in these calculations, and a Poisson ratio of 0.26 has been adopted (Kim *et al.*, 2001 ▸; Hopcroft *et al.*, 2010 ▸). This model accurately predicts the sagittal focal points expected for these crystals at various energies. Beam size was subsequently determined from the FWHM of the simulated beam profiles when the beam was at sagittal focus. Beam divergence was determined geometrically from the beam size at sagittal focus and the beam size at the monochromator crystal. X-ray beam flux and energy bandpass are determined automatically by *xrt* during ray generation. Ray generation in *xrt* is performed through pulses where each photon is assigned an energy within the energy range of the source. The beam flux is then determined by monitoring the number of photons that successfully reach the detector position over the duration of the pulse. Energy bandpass is subsequently calculated from the FWHM of the energy distribution of photons that have reached the detector position.

## 4. Beamline capabilities and uses

**Figure 7:**
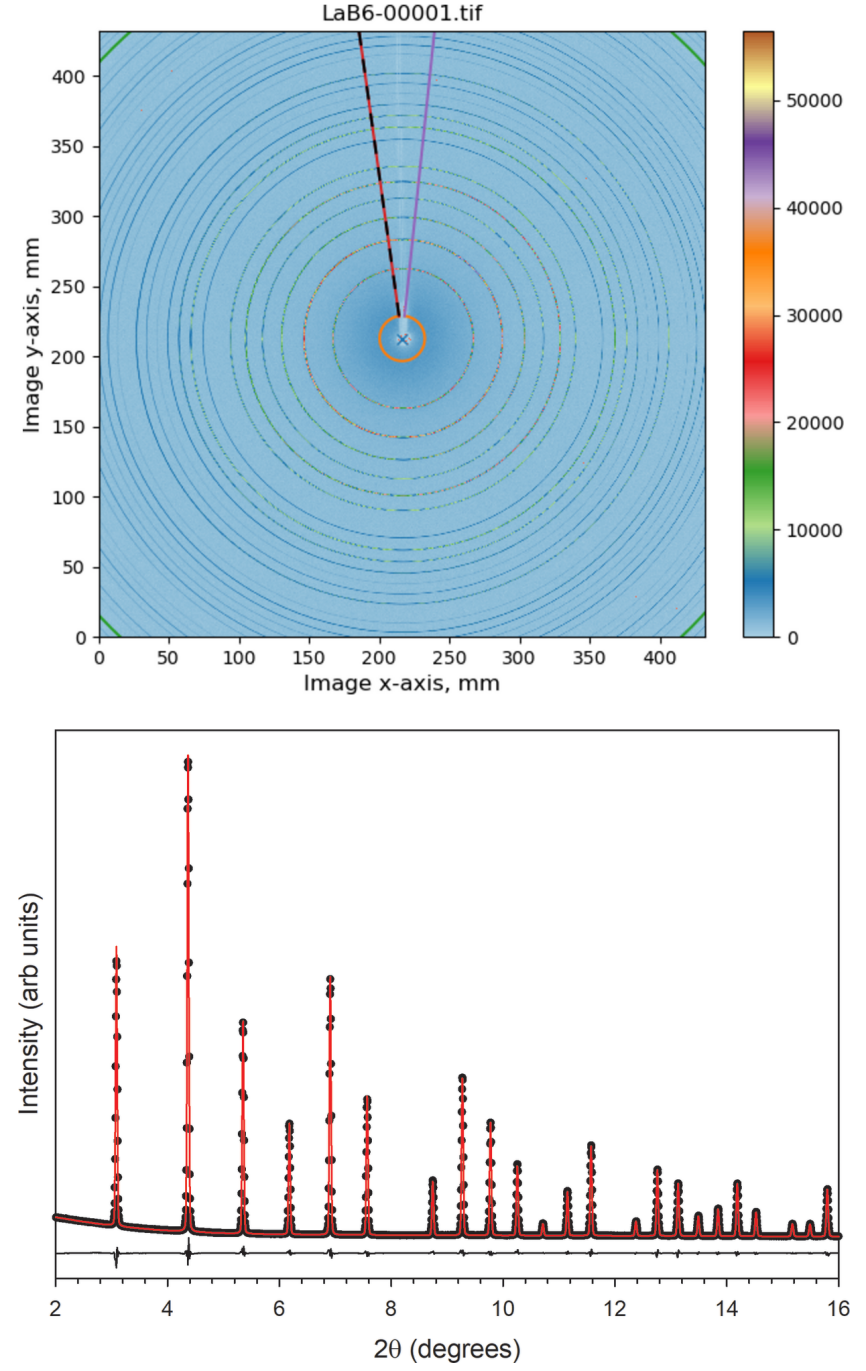
Top: 2D diffraction pattern of LaB_6_ collected using 55.32 keV X-rays with the Varex detector placed 923 mm away from the sample. Bottom: a Rietveld refinement of the integrated LaB_6_ data. Black circles are the data points, the red line is the fit, and the difference is shown beneath. The *R*_wp_ for the fit is 1.91.

The two detectors currently available are a Varex XRD 4343CT and a Dectris Eiger2 4M. As nearly the entire Debye-Scherrer ring is collected and integrated (except where the shadow of the rod holding the beamstop is located), very high statistic data can be collected very rapidly. Complete powder diffraction patterns with low noise can usually be collected in just a few seconds and often in just tenths of a second. This makes the beamline well suited for rapidly acquiring data on large numbers of samples, monitoring fast chemical reactions, or collecting temperature dependent data using a fine temperature increment (King, 2020 ▸). The two-dimensional information given by the area detector can also be used for texture analysis. Examples of a typical two dimensional diffraction pattern and a Rietveld refinement of the radially integrated data are shown Fig. 7 ▸.

Many experiments have used the higher energies of the beamline to collect powder diffraction data on thick samples or devices. Examples include monitoring phase changes in high strength steels during rapid heating and cooling (Ghosh *et al.*, 2022 ▸; Singh *et al.*, 2022 ▸; Wang *et al.*, 2021 ▸) and tensile measurements on pipes or sheets of metal (Kong *et al.*, 2023 ▸). Another popular use is monitoring of phase changes within batteries. Either prototype or fully functional commercial battery pouch cells or coin cells can be placed on the beamline and the composition can be studied at various locations in the battery as a function of cycling (Bond *et al.*, 2024 ▸).

The beamline is also capable of performing high pressure experiments using a diamond anvil cell (DAC) (Ukoji *et al.*, 2024 ▸; Guan *et al.*, 2024 ▸). Typically, an energy around 35 keV is used for such experiments, as it has the proper penetration to transmit through the cell and provides data in a useful *d*-spacing range that matches the exit angle of the cell. A setup containing a collimator and pinhole is used to reduce the beam size to a diameter as small as 20 µm. A motorized stage stack is used to hold and center the DAC. Alignment is achieved through an active beamstop which has a diode embedded in it to monitor the flux transmitting through the components.

**Figure 8:**
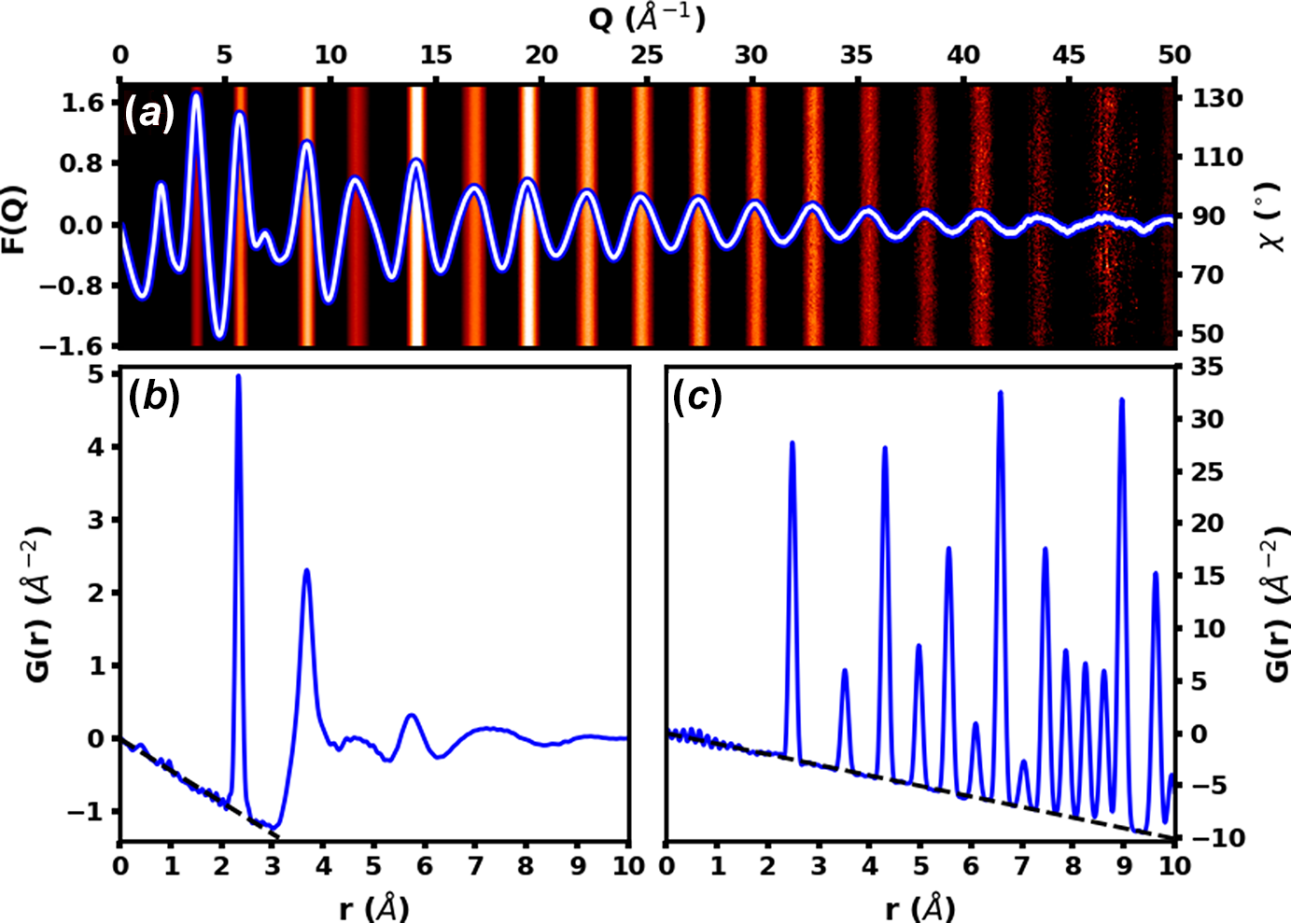
(*a*) A one-dimensional reduced total scattering structure function *F*(*Q*) and a two-dimensional *QF*(*Q*) of amorphous selenium where white/red indicates positive intensity and black indicates negative intensity. (*b*) A PDF of amorphous selenium generated using a *Q*_max_ of 50 Å^−1^. (*c*) A PDF of Ni metal generated using a *Q*_max_ of 42.5 Å^−1^. Data were collected on a Perkin Elmer detector using (*a*, *b*) 72 keV and (*c*) 60 keV radiation.

**Figure 9:**
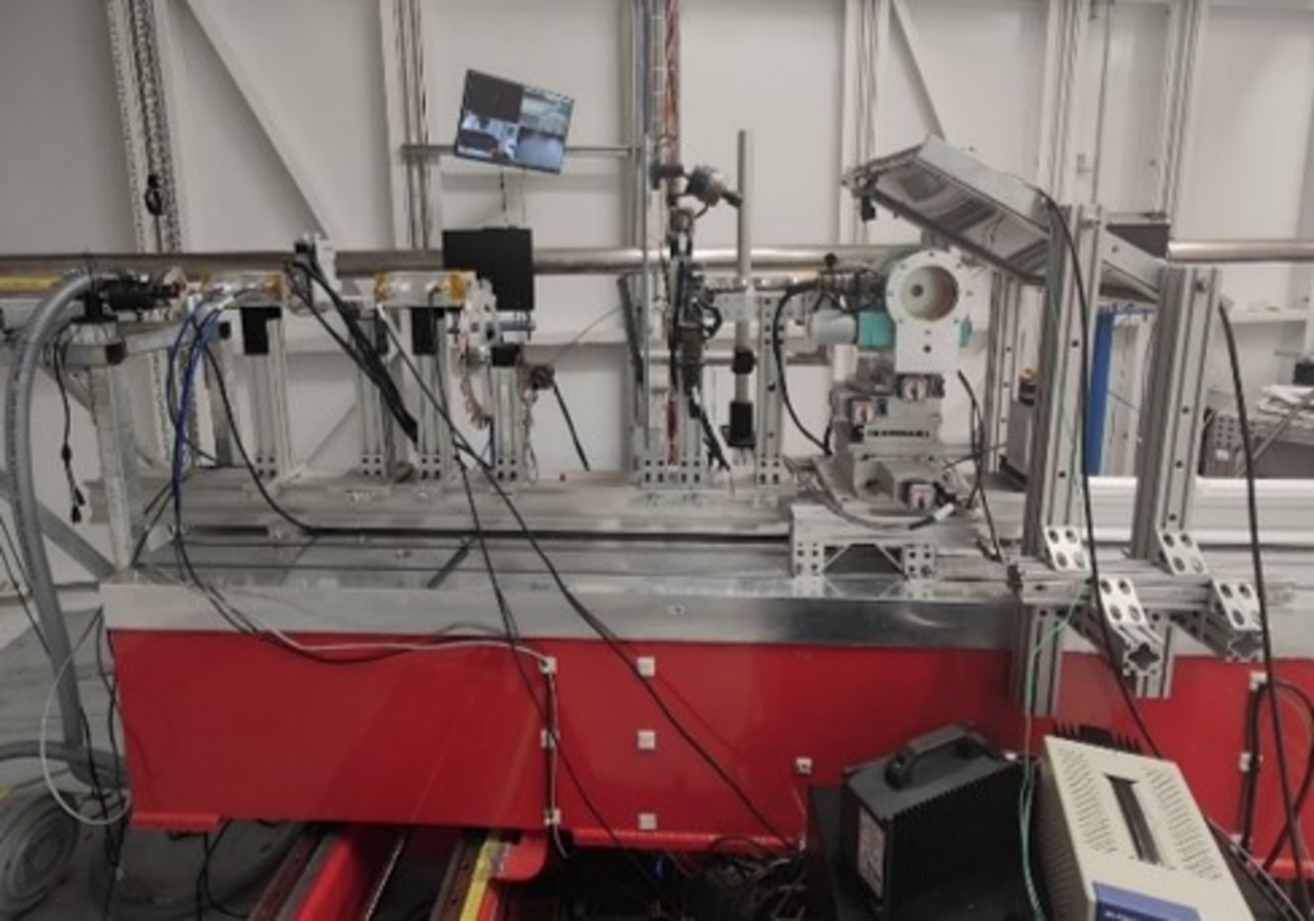
Photograph of inside the BXDS-HE experimental hutch with the detector arranged in tilted geometry. The detector is inclined approximately 60° from normal incidence geometry.

High energy X-rays also allow access to high *Q* data, which is essential for producing pair distribution functions (PDFs) with good real space resolution. One of the main uses of this beamline is the collection of high quality PDF data. The PDF gives the distribution of interatomic distances in a material and is an excellent probe of the short and medium range structural features. It can be applied to almost any area of research, including poorly crystalline minerals (King *et al.*, 2020 ▸), single atom catalyst (Chen *et al.*, 2022 ▸; Xia *et al.*, 2021 ▸), magnetic materials with site disorder (Lozano-Gorrín *et al.*, 2021 ▸), glasses (Singh *et al.*, 2021 ▸; Wang *et al.*, 2022 ▸), and nanoparticles (Sulaiman *et al.*, 2022 ▸), to name a few. On this beamline, PDF data are most commonly collected using 60 keV X-rays, which allows a *Q*_max_ around 27 Å^−1^ with the large 43 cm × 43 cm Varex detector placed close to the sample. Using higher energies, it is possible to collect data well above 30 Å^−1^. A new methodology for PDF data collection has recently been developed on this beamline which uses the area detector in an inclined geometry to cover a 2θ range 0–90°. This geometry has several advantages such as extending the accessible *Q* range and an improved signal to noise ratio at higher scattering angles (Burns *et al.*, 2023 ▸). Some examples of data collected in this configuration are shown in Fig. 8 ▸ and a photograph of the setup is shown in Fig. 9 ▸.

A number of different sample environments are available. For variable temperature experiments a cryostream can precisely control the temperature between 80 and 500 K and a capillary furnace can heat as high as 1473 K while also flowing gas. A gas loading system is available for high pressure experiments. Special battery holders have been designed to use with battery cyclers. The beamline is equipped with a 10 kN stress rig integrated with an infra-red furnace to accommodate various types of strain experiments.

## 5. Summary

A new high energy wiggler beamline at the Canadian Light Source is available for conducting high energy diffraction, high pressure diffraction, and pair distribution function studies. The beamline uses a single bent Si crystal monochromator and a translation table in the hutch to follow the beam. The flux, bandwidth, divergence, and focus of the beam have been measured and compare well with theoretical values determined from ray tracing calculations. The high energies are suitable for *in situ*/*operando* experiments under extreme conditions with large samples and environments for new alloys, catalysis, batteries, among others. Since opening for users in 2020, the user’s community has been rapidly growing, taking advantage of these new beamline capabilities in numerous research areas.

## Figures and Tables

**Figure 5:**
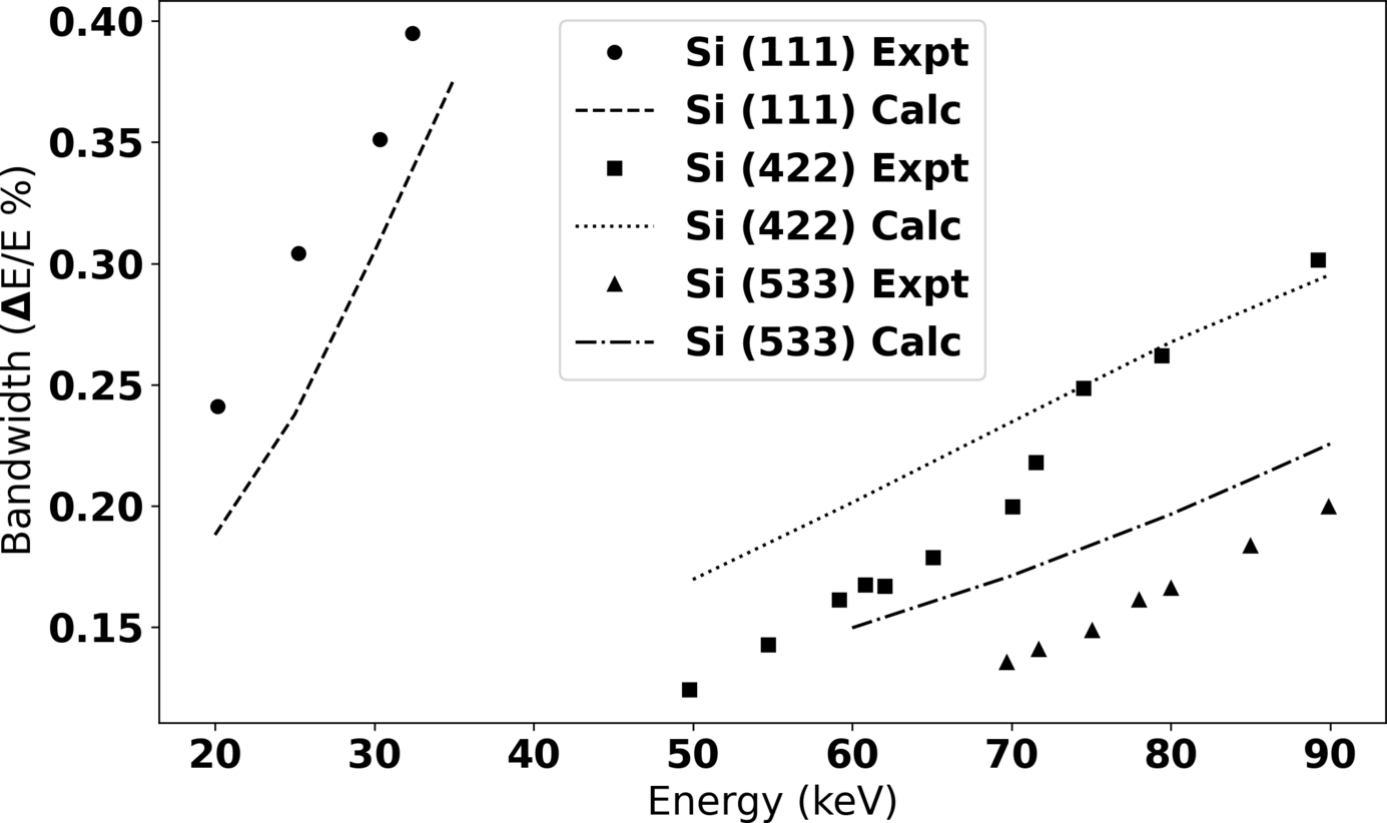
Experimentally measured energy bandwidths using rocking curves of Si (333) and (777) reflections. These values are over-plotted with simulated values from ray tracing calculations.

**Table 1:** HE beamline flux and beam size measurements at three energies

Energy (keV)	Vertical FWHM (µm)	Horizontal FWHM (mm)	Flux density (photons s^−1^ mm^−2^)
30	31	2.05	1.4 × 10^14^
60	19	2.23	1.1 × 10^13^
80	25	2.39	3.0 × 10^11^
